# Cped1 promotes chicken SSCs formation with the aid of histone acetylation and transcription factor Sox2

**DOI:** 10.1042/BSR20180707

**Published:** 2018-09-14

**Authors:** Chen Zhang, Fei Wang, Qisheng Zuo, Changhua Sun, Jing Jin, Tingting Li, Man Wang, Ruifeng Zhao, Xinjian Yu, Hongyan Sun, Yani Zhang, GuoHong Chen, Bichun Li

**Affiliations:** Key Laboratory of Animal Breeding Reproduction and Molecular Design for Jiangsu Province, College of Animal Science and Technology, Yangzhou University, Yangzhou 225009, China

**Keywords:** chicken, differentiation, Histone acetylation, Spermatogonial stem cell, Sox2

## Abstract

Spermatogonial stem cells (SSCs) may apply to gene therapy, regenerative medicine in place of embryonic stem cells (ESCs). However, the application of SSCs was severely limited by the low induction efficiency and the lack of thorough analysis of the regulatory mechanisms of SSCs formation. Current evidences have demonstrated multiple marker genes of germ cells, while genes that specifically regulate the formation of SSCs have not been explored. In our study, cadherin-like and PC-esterase domain containing 1 (Cped1) expressed specifically in SSCs based on RNA-seq data analysis. To study the function of Cped1 in the formation of SSCs, we successfully established a CRISPR/Cas9 knockout system. The gene disruption frequency is 37% in DF1 and 25% in ESCs without off-target effects. Knockout of Cped1 could significantly inhibit the formation of SSCs *in vivo* and *in vitro*. The fragment of −1050 to −1 bp had the activity as Cped1 gene promoter. Histone acetylation could regulate the expression of Cped1. We added 5-azaeytidi (DNA methylation inhibitors) and TSA (histone deacetylase inhibitors) respectively during the cultivation of SSCs. TSA was validated to promote the transcription of Cped1. Dual-luciferase reporter assay revealed that active control area of the chicken Cped1 gene is −296 to −1 bp. There are Cebpb, Sp1, and Sox2 transcription factor binding sites in this region. Point-mutation experiment results showed that Sox2 negatively regulates the transcription of Cped1. Above results demonstrated that Cped1 is a key gene that regulates the formation of SSCs. Histone acetylation and transcription factor Sox2 participate in the regulation of Cped1.

## Introduction

Spermatogonial stem cell (SSCs) is the only adult stem cell in the body that can transmit genetic information to their offspring. They have the potential to be induced to multiple cells *in vitro*, which may replace embryonic stem cells (ESC) for gene therapy and genetic research without ethical issues. However, the key gene regulating the formation of spermatogonial stem cells and its mechanism remains unclear. Therefore, inefficient differentiation of SSCs *in vitro* and poor reproducibility make it difficult to reach the need of research and practice. Moreover, there are fewer reports in poultry. In summary, further exploring the regulation genes of SSCs and its molecular mechanisms are beneficial to establish a mature inducing system *in vitro* for SSCs.

In 1994, Brinster et al. [1] successfully used SSCs for allogeneic transplantation to produce sperm, which verified the presence of SSCs in donor spermatogonia. However, the scarce amount of SSCs and the lack of specific markers still needed to be solved. The researchers tried to search for the surface markers of SSCs via the mass screening. Integrin α6 and integrin β1 were found for the surface markers of SSCs by forming laminin receptors [2]. Moreover, thymine-1 (Thy-1), CD9, and CD24 were also identified in SSCs through binding immunity and cell transplantation experiments [3–6]. With further research, the problems are constantly highlighted: the surface markers are often not specifically expressed genes in SSCs and cannot be accurately used for cell screening. The root of the problem lies in how to deeply understand the specific molecular mechanisms of SSCs, increase the induction efficiency of SSCs in vitro, and meet the actual application requirements. Therefore, many researchers have focused on exploring gene regulation for SSCs self-renewal and formation at different levels. The self-renewal and proliferation of SSCs depend on GDNF [7]. Oatley et al. [8] found that GDNF could up-regulate Bcl6b or other transcription factors by SFK signaling to drive SSC self-renewal. Ets5 plays an important role in regulating the self-renewal of SSCs (citation). Several researchers found that the absence of ERM made SSCs gradually decrease and eventually deplete [9–12]. Dann et al. [13] suggested that RA can trigger spermatogonial differentiation by direct or indirect down-regulation of OCT4 and promyelocytic zinc finger (PLZF), revealing that OCT4 and PLZF can maintain SSCs in an undifferentiated state.

Recent studies only focus on the regulation mechanism of the self-renewal and proliferation of SSCs. However, few researches were performed on the genes regulating the formation of SSCs. Moreover, the regulation mechanism has not been elaborated. Furthermore, it is hard to obtain a large amount of SSCs. Currently, our team are dedicated to efficiently obtain SSCs by studying the mechanism of SSCs via chicken model. Our previous studies suggest that Cped1 that only exists in poultry is specifically expressed in SSCs via RNA-seq analysis. It is reasonable to speculate that Cped1 may play an important role in the formation of SSCs.

In order to reveal the regulation of Cped1 in the formation of chicken SSCs, we knocked out and overexpressed Cped1 *in vitro* and *in vivo*. These results implied that Cped1 could facilitate SSCs formation under the control of histone acetylation and transcription factor Sox2. Our study could lay a solid foundation for exploring the genetic regulatory mechanisms in the formation of chicken SSCs.

## Materials and methods

### Materials

Dulbecco’s modified eagle medium (DMEM) and fetal bovine serum (FBS) were obtained from Gibco (Carlsbad, CA, U.S.A.). Human stem cell factor, basic fibroblast growth factor, retinoic acid (RA), and murine leukemia inhibitory factor were acquired from Sigma-Aldrich (Saint Louis, MO, U.S.A.). TRNzol, FastQuant-RT kit, and SuperReal premix color kit were obtained from TIANGEN (Beijing, China). Fugen was from Promega (Madison, WI, U.S.A.). Antibodies specific to the following proteins were integrinα6 (Millipore, temecula, CA, U.S.A.; dilution ratio 1:100), integrin β1 (Millipore, temecula, CA, U.S.A.; no. MAB1378; dilution ratio 1:100), goat anti-Rat IgG (Proteintech, Chicago, Illinois U.S.A.; no. SA00003-11; [FITC] labeled; dilution ratio, 1:100), and goat anti-rabbit IgG (Proteintech, Chicago, Illinois, U.S.A.; no. SA00008-9; [PE] labeled; dilution ratio, 1:100).

## Method

### Isolation culture of ESCs and SSCs

ESCs were collected from embryo disc purchased from the farm of poultry research institute (Chinese Academy of Agricultural Science, China). PGCs and SSCs were collected from reproductive crest and testicles in 4.5 d and in 18.5 d after hatching at 37°C and 60% RH (relative humidity) [14,15].

### Generation of constructs

The full-length Cped1 gene was amplified with the sequence released in NCBI as the template. Primers applied were: F(Kpn1): CGGggtaccAGTGAGGACAGCACGTTGGCC; R(EcoR1): CCGgaattcTGCAGCCAATCAAGGAAGCGT. The product was cleaved and ligated into the corresponding sites of pcDNA3.0 plasmid and verified via sequencing. The Cped1-KO vector was constructed in Genomeditech (Shanghai, China). Different fragments of Cped1 promoter were amplified to be ligated into pGL3-basic vector. Primers applied were shown in Table 1.

**Table 1:** Primers of different fragments

Fragments	Primer
−1050 to −1 bp	F: 5′ CCGCTCGAGAATGGCATCTTCTGGGTG 3′
−860 to −1 bp	F: 5′ CCGCTCGAGCATGTGCAAATTGACCG 3′
−670 to −1 bp	F: 5′ CCGCTCGAGATATGAGGCAGTGTAGGA 3′
−480 to −1 bp	F: 5′ CCGCTCGAGGGGAAGTAGTAACTAAGACC 3′
−296 to −1 bp	F: 5′ CCGCTCGAGAAACCTGCTTCCTTTCACA 3′

### Cell culture and cell transfection

The ESCs were cultured in DMEM containing 10% FBS, human stem cell factor, basic fibroblast growth factor, human insulin-like growth factor, and murine leukemia inhibitory factor incubated at 37°C, 5% CO_2_ with saturated humidity. The second generation ESCs were inoculated into the 24-well cell culture cluster (including glass coverslips; 10^5^ cells/well) under RA induction (Supplementary Figure S1). Culture medium was replaced every 2 d, cell morphology was observed by inverted microscope, and cell samples were collected for subsequent experiments. Plasmid vectors for transfection were prepared using FuGene and transfected into ESCs at the ratio of plasmid:FuGENE (density/volume) = 1:3.

### Luciferase reporter assays

The Dual-Luciferase Reporter Assay System was used to monitor the promoter activity of Cped1 fragments. DF1 cells were co-transfected with 1000 ng plasmids of SV40 and respective reporter constructs (1:30). Firefly and Renilla luviferase activity were measured after 48 h using BioTek. The firefly luciferase signal was normalized against the Renilla signal and against the empty vector PGL3-basic as control.

### qRT-PCR

The total RNA of collected PGCs and SSCs from *in vivo* and *in vitro* samples was extracted by TRNzol reagent. cDNA was synthesized by TIANGEN reverse-transcription kit. The primers used were listed in Table 2. The PCR instrument for qRT-PCR was ABI PRISM 7500 (Applied Biosystems, Carlsbad, California). The reaction mixture for qRT-PCR consists of 2 μl of cDNA, 10 μl of SuperReal premix, 0.6 μl of forward primers (10 μmol/l), 0.6 μl of reverse primers (10 μmol/l), and 0.4 μl of 50× Rox. Total volume of the reaction mixture was adjusted to 20 μl with ddH_2_O. Subsequently, PCR reaction was achieved based on the two-step procedure (95°C for 15 min; 95°C for 10 s, 60°C for 32 s), and procedure was repeated 40 times (*n* = 3). β-Actin was used as an internal control gene. The relative expression of each gene was calculated by 2−^ΔΔ*C*^_^t^_method.

**Table 2:** qPCR primers of related genes

Gene name	Primers Sequence (5′-3′)		Size (bp)
*CPED1*	F: CAGCGACACACATTGCTTCT	R: GCAGGAGGTGCGTACAGAAT	120
*Nanog*	F:TGGTTTCAGAACCAACGAATGAAG	R:TGCACTGGTCACAGCCTGAAG	180
*Sox2*	F:GAAGATGCACAACTCGGAGATCAG	R:GAGCCGTTTGGCTTCGTCA	100
*C-kit*	F:GCGAACTTCACCTTACCCGATTA	R:TGTCATTGCCGAGCATATCCA	150
*Cvh*	F:AGGAGGACTGGGACACG	R:GCCTCTTGATGCTACCG	1646
*Dazl*	F:TGTCTTGAAGGCCTCGTTTG	R:CATATCCTTGGCAGGTTGTTGA	138
*Stra8*	F:CCACGGCTATTTCACACCTCTG	R:GCTCTTGGCAAGCATCCGTA	114
*Integrin α6*	F:GAAACCCGGGATATCATTGG	R:CAGCAACACCTTGCTGACAG	140
*Integrin β1*	F:TGTTTGTGGGGACCAGATTG	R:CCAGGTGACATTTCCCATCA	120
*β-Actin*	F:CAGCCATCTTTCTTGGGTAT	R:CTGTGATCTCCTTCTGCATCC	164

### Immunofluorescence

The glass coverslips were collected after 12 d. Paraformaldehyde (4%) was used for fixing for 30 min and the PBS was rinsed twice. The glass coverslips were incubated with 10% BSA-PBS reagent at 37°C for 2 h. The primary antibodies were added onto the corresponding samples. The mixture was incubated at 37°C for 2 h, and then kept overnight at 4°C. After three times rinse by PBST, FITC-labeled or PE-labeled secondary antibody was diluted 100 times, and added to the mixture and then incubated at 37°C in the dark for 2 h. After three times rinse by PBST, the coverslips were dropped in 5 ng/μl DAPI for 15 min. After washing, the glass coverslips were examined with confocal microscopy (OLYMPUS) and photographed.

### Flow cytometry analysis

PGCs and SSCs were collected *in vivo* and *in vitro*. The germ cells were labeled with different antibodies. Flow cytometry was used to analyze the cell positive rate.

### Paraffin section

Chicken embryo (5.5 d) was fixed 18–24 h, then in 70%, 80%, 90%, 95%, and 100% alcohol dehydration for half an hour and be transparent in xylene for 10 min. After immersing in wax for 1 h, embedding, waxing and slicing were performed. The embryo was dewaxed and rehydrated for the next PAS staining.

### Data analysis

SPSS software was used to process data. The differences between the data groups were analyzed by single factor analysis of variance. LSD method was used to compare the data. *P*<0.05 was used to define statistical significance. The results are expressed as an average.

## Result

### Cped1 is specifically expressed in chicken SSCs

**Figure 1:**
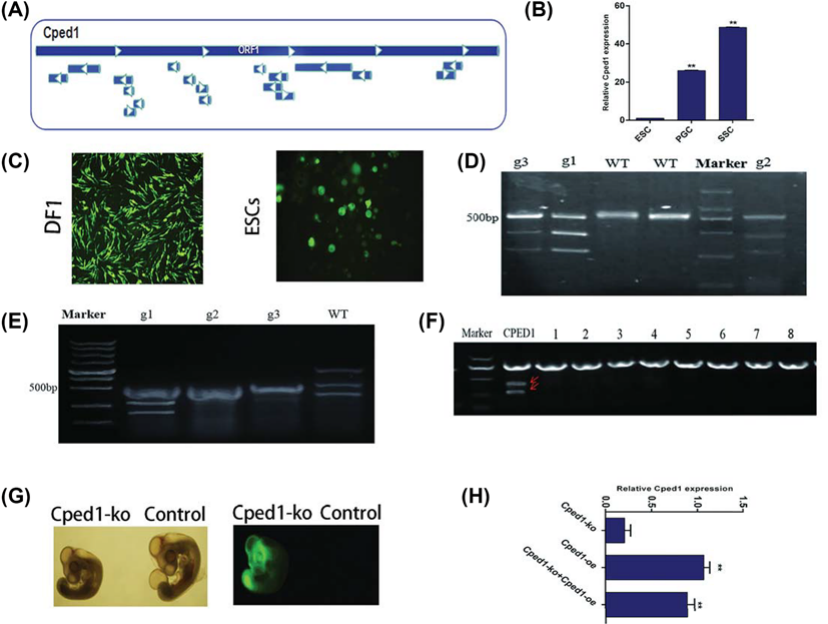
Cped1-ko and Cped1-oe were successfully constructed (**A**) Bioinformatics analysis showed that Cped1 contains 38 open reading frames. (**B**) The expression of Cped1 was detected by qrt-PCR in ESCs, PGCs, and SSCs. (**C**) DF1 (100×) and ESCs (200×) were transfected by Cped1-ko. (**D**) DF1 was collected to be examined by T7E1 restriction. (**E**) ESCs was collected to be examined by T7E1 restriction. (**F**) Different off-target sites were detected by T7E1 restriction. (**G**) After injection by Cped1-ko, chicken embryo showed positive eGFP under visual fluoroscopy. (**H**) The expression of Cped1 was detected by qrt-PCR in different groups; **P<0.01. Data are representative of at least three independent experiments.

In order to explore the key genes in the formation of chicken SSCs and complete the regulation mechanism of SSCs, we found Cped1 was specifically expressed in SSCs by RNA-seq of chicken ESCs, PGCs, and SSCs [16]. Bioinformatics analysis revealed that Cped1 contains 38 open reading frames (Figure 1A), encoding 1058 amino acids, which may be essential in SSCs. Through expression profiling analysis, Cped1 have the highest expression in SSCs compared with ESCs and PGCs (Figure 1B). Above results suggest that Cped1 is specifically expressed in chicken SSCs and may be a potential gene for regulating the formation of SSCs.

### Cped1 promotes the formation of SSCs during chick embryo development

To further explore whether Cped1 can affect the formation of SSCs during chicken embryo development, a CRISPR/Cas9 knockout vector was successfully constructed. We designed three gRNA target sites (named g1, g2, and g3) (Table 3). The result of T7E1 digestion showed that the Cped1-KO system (g1) could knockout Cped1 gene and had different knockout activity between DF1( 37% ) and ESCs (25% ) (Figure 1C–E). Also, we predicted off-target site of Cped1 and digested by T7E1 to study the off-target problem of the CRISPR/Cas system (Table 4). There was no off-target phenomenon in Cped1-DF1 cells (Figure 1F). After the injection, it was found that the Cped1-KO system could stably exist in chick embryos and the embryos could express fluorescence (Figure 1G). Cped1-OE recombinant plasmid transfected DF1 cells and qRT-PCR showed the high expression of Cped1. Then, Cped1-KO and Cped1-OE recombinant plasmids were co-transfected. We found that the expression of Cped1 was increased compared with transfecting Cped1-KO. However, transfecting Cped1-OE alone, the expression of Cped1 increased compared with co-transfecting Cped1-KO and Cped1-OE (0.1<*P*<0.5) (Figure 1H).

**Table 3:** sgRNA target site nucleotide sequence

Name	gRNA sequence	PAM
gRNA1 (g1)	GCGACAAAGCCTGCGGCACC	TGG
gRNA2 (g2)	GTTGGTCATGAGGGGAGTCA	TGG
gRNA3 (g3)	TGGTTTGTGGGCACGGCCCC	TGG

**Table 4:** Target primers used in off-target efficiency detection

NO.	Gene ID	Primer
1	XM_009084842.1	F:5′ TAAAGCCCGCTTTGGCATTT 3′
		R:5′ CACAGAGCTCAGACTTCCAA 3′
2	XR_718405.1	F:5′ GGAAAGCACTCAGCAGCAGG 3′
		R:5′ TGTTAACTATTGCTCAGCACTCT 3′
3	XM_008173115.1	F:5′ GGAAAAACACTCCTTTGTCTTT 3′
		R:5′ TCCGTATTGTGCTAGGATGC 3′
4	XM_002739438.2	F:5′ ATTCACTCGTATTCGCAGATGT 3′
		R:5′ GAAATAAGTTGCCACTCCAT 3′
5	CP003880.1	F:5′ TGCTCAAACACCCGCCAGTC 3′
		R:5′ CTTGACCATGTCCGCCACGA 3′
6	CP003244.1	F:5′ TGGCACCTTCCCGTCCAAGC 3′
		R:5′ CAGCAGGGCATTAAAGGTGT 3′
7	CP018202.1	F:5′ ATTCGGTCAGCAGCGTTTCA 3′
		R:5′ CGCTCCGAATGCAGGTAGAC 3′
8	CP006835.1	F:5′ CGAACAATTCCGATTGCAGTATC 3′
		R:5′ AGACGTTGGAGGCGTGGTTCAT 3′

**Figure 2:**
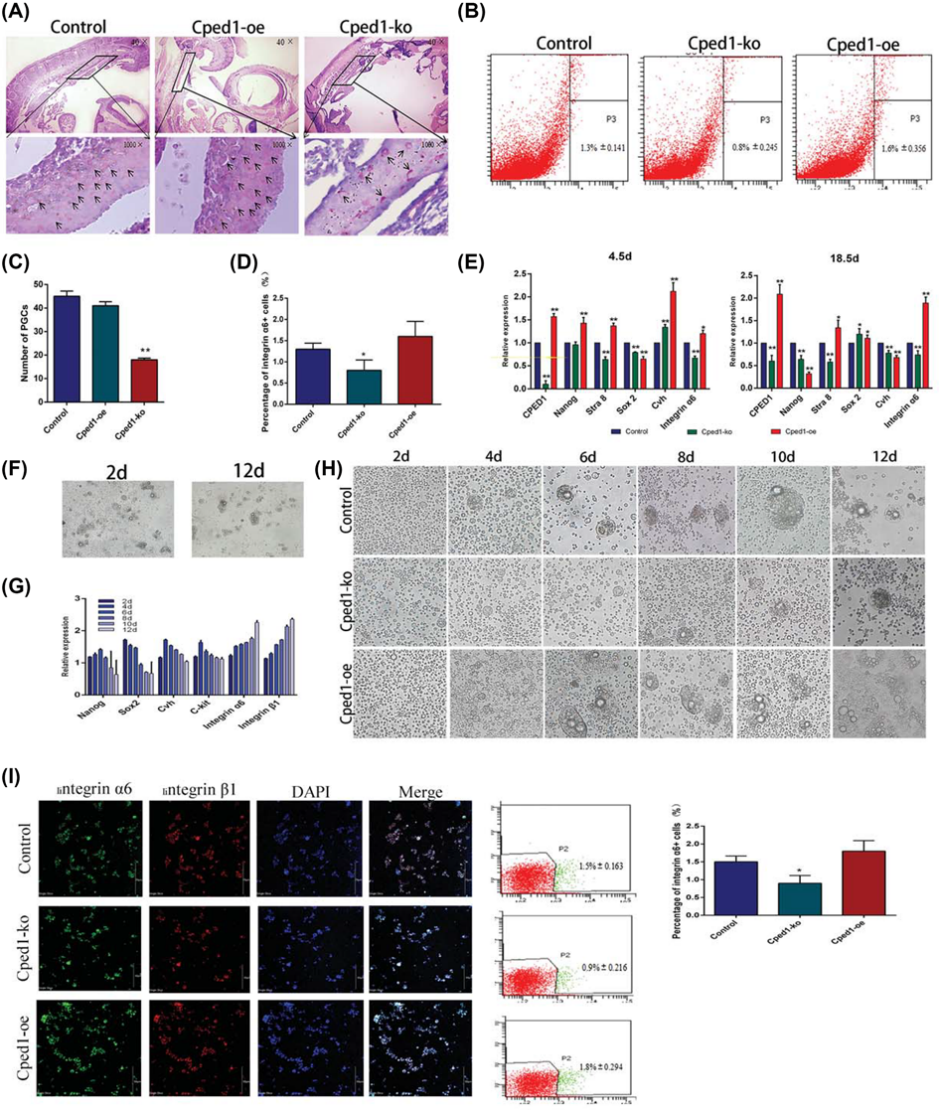
Cpend1 promotes the formation of SSCs *in vivo* and *in vitro* (**A** and **C**) PAS staining showed the number of SSCs. (**B** and **D**) Flow cytometry showed the number of SSCs. (**E**) The expression of marker genes was detected in 4.5 and 18.5d by qrt-PCR. (**F**) In RA induction model, cells were observed in 2 and 12 d (400×). (**G**) Marker genes were detected in 2, 4, 6, 8,10, and 12 d by qrt-PCR. (**H**) After transfected by Cped1-ko or Cped1-oe, cells were observed in 2, 4, 6, 8, and 10, and 12 d (400×). (**I**) SSC-like were respectively detected by immunofluorescence and flow cytometry analysis (400×); *P<0.05 and **P<0.01. Data are representative of at least three independent experiments.

Besides, we made use of knockout and overexpression vector to identify the function of Cped1 in chick embryos. After knocking out Cped1, the amount of PGCs was significantly reduced (*P*<0.01) in 4.5 d, and they mainly located in the head and tail of genital crest while less in the middle. After overexpression of Cped1, PGCs were distributed throughout the genital crest, and the amount of PGCs was evenly distributed, where there is no difference with normal hatching chicken embryos (Figure 2A,C). Flow cytometry showed that under knockout Cped1, the amount of SSCs was significantly reduced (*P*<0.01) while under overexpressed Cped1, they are not changed. Although overexpression of Cped1 could not increase the amount of SSCs, it can improve the expression of germ cell marker genes at the transcription level (Figure 2B,D) These results predicted that knocking out Cped1 may inhibit the formation of PGCs, which indirectly affects the formation of SSCs. Moreover, qRT-PCR showed that the markers of Stra8 and integrin α6 were both significantly reduced (*P*<0.01) under Cped1 knockout system in 4.5 d while overexpression of Cped1, Cvh was significantly increased (*P*<0.01). Stra8 and integrin α6 exerted expression deficiency in 18.5 d after knockout Cped1 (*P*<0.01). Under overexpressed Cped1, Nanog significantly decreased and integrinα6 was significantly up-regulated (*P*<0.01) (Figure 2E). Above results suggested that Cped1 can promote the formation of SSCs during chick embryo development.

### Cped1 promotes the formation of SSCs-like during RA induction

We have found that Cped1 could promote the formation of SSCs *in vivo*. To further determine the function of Cped1, we successfully constructed an RA induction model [17]. After adding RA in the medium to 10^−5^mol/l, we found small embryoid bodies were observed on second day and became bigger. Then SSC-like appeared on the 12th day (Figure 2F). The expression of stem cell marker Nanog increased from second to sixth day, and began to decrease continuously on eight day, while Sox2 decreased continuously from second day. On the contrary, Cped1 continuously increased (Figure 2G).

**Figure 3:**
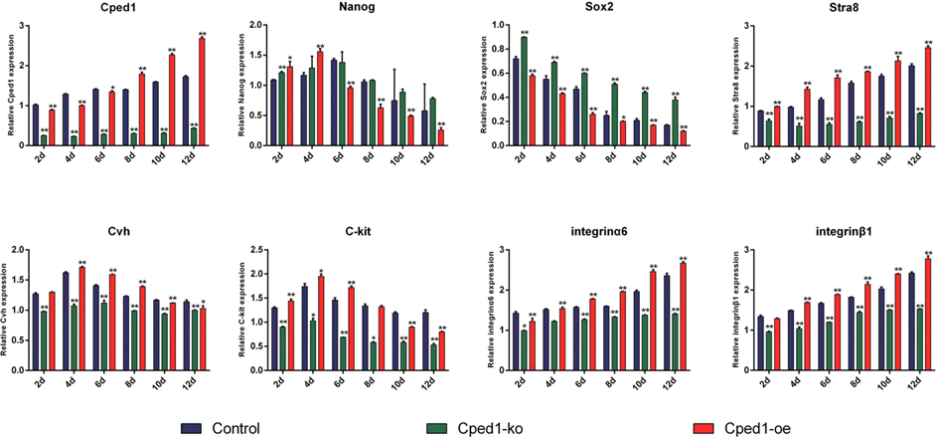
Cped1 influences the expression of marker genes in different stages qrt-PCR was used to detect the expression of marker genes in 2, 4, 6, 8, 10, and 12 d after transfecting Cped1-ko and Cped1-oe; *P<0.05 and **P<0.01. Data are representative of at least three independent experiments.

During the induction of RA, we used knockout and overexpression vector to explore the function of Cped1 in the formation of SSCs. After knockout Cped1, embryoid bodies emerged on 2nd day while SSC-like did not appear on 12th day. Overexpressing Cped1, we found that the edges of cells became nicked on 6th day and SSCs-like appeared on 8th day (Figure 2H). These results indicated that Cped1 may promote the formation of SSCs *in vitro*. We subsequently detected the expression of pluripotency and reproduction markers. Nanog and Sox2 have no changes after knockout Cped1 while Cvh, C-kit, Stra8, integrinα6 and integrin β1 were significantly reduced (*P*<0.01). By overexpressing Cped1, Nanog and Sox2 became decreased (*P*<0.01) while Cvh, C-kit, integrin α6 and integrin β1 were not change (Figure 3). Above results demonstrated knockout Cped1 could inhibit the formation of SSC-like *in vitro*.

In order to detect the expression of markers at the protein level, we used immunochemical assay and found integrin α6^+^ and integrin β1^+^ cells were significantly reduced on tenth day after knockout Cped1. Overexpression of Cped1 did not influence the formation of SSC-like (Figure 2I). Flow cytometry showed that integrin α6^+^ cells decreased after knockout Cped1 (0.9%) and there was no difference after overexpression Cped1 (oe: 1.8%; blank: 1.5%) (Figure 2I). Therefore, Cped1 could promote the formation of SSC-like under RA induction system.

### Histone acetylation together with Sox2 regulates the expression of Cped1

**Figure 4:**
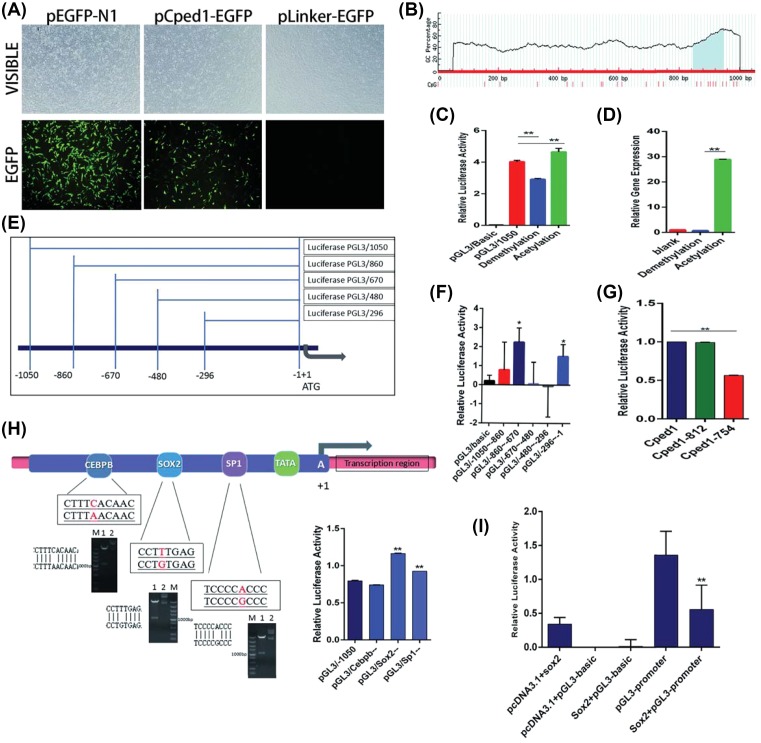
Histone acetylation and transcription factor Sox2 regulate the expression of Cped1 (**A**) After transfecting pCped1-EGFP, DF1 showed the positive eGFP (100×). (**B**) Bioinformatics analysis showed the CPG island in the promoter of Cped1. (**C**) After adding 5-azaeytidi and TSA in DF1, dual-luciferase report showed the active of Cped1 promoter. (**D**) After adding 5-azaeytidi and TSA in SSCs, the expression of Cped1 was detected by qrt-PCR. (**E**) Different fragments of Cped1 promoter. (**F**) Dual-luciferase report revealed the active of different fragments of Cped promoter. (**G**) Two regions pGL3/-860 to -670 and PGL3/-296 to -1 were separately deleted. Dual-luciferase report showed the active of mutated fragment. (**H**) After mutating transcription factor binding site, the active of promoter was detected by dual-luciferase report. (**I**) The active of promoter was detected by dual-luciferase report after overexpression Sox2; *P<0.05 and **P<0.01. Data are representative of at least three independent experiments.

By detecting the expression of Cped1 in different cells, we found that Cped1 was expressed specifically in SSCs. The differences in temporal may be caused by the promoter of gene. To clarify the expression mechanism of Cped1, we cloned the predicted promoter (−1050 to −1 bp) and replaced the pEGFP-N1 promoter. Fluorescence was observed by transfecting DF1, which means fragment of −1050 to −1 bp had the activity as Cped1 gene promoter (Figure 4A).

Gene promoter regions are often affected by epigenetic modifications that may influence the transcription of gene. Bioinformatics analysis revealed that Cped1 has CpG islands in the promoter region (Figure 4B). To detect whether Cped1 is modified by DNA methylation, we added 5-azaeytidi in DF1 cells (Figure 4C). Dual-luciferase report revealed that after demethylation, Cped1 activity was significantly reduced, suggesting that Cped1 may not be regulated by DNA methylation. To determine whether DNA methylation could regulate the expression of Cped1 in SSCs, we utilized qRT-PCR and found there was no significant change in Cped1 expression after DNA demethylation (Figure 4D). Above results indicated that the CpG island enriched in the Cped1 promoter region without DNA methylation regulation. We speculated the number of CpG sites in the promoter region is insufficient to cause long-distance gene suppression. However, we found the activity of Cped1 promoter increased in DF1 cell with TSA and the expression of Cped1 significantly increase in SSCs (Figure 4C,D). These results confirmed that Cped1 was regulated by histone acetylation.

In addition to DNA methylation and histone modifications, gene transcriptional activity may also be regulated by transcription factors. Two regions pGL3/-860 to -670 and PGL3/-296 to -1 with high activity of promoter were determined (Figure 4E,F). Then we separately deleted fragments and found that the fragment of -296 to -1 bp was the active control area of Cped1 (Figure 4G). The predicted transcription factors Cebpb, Sp1, and Sox2 were mutated respectively. Dual-luciferase report showed that Cebpb had no significant effect on the transcription of Cped1, Sp1 positively regulated the transcription of Cped1 while Sox2 negatively regulated the transcription of Cped1 (Figure 4H). Since sp1 ubiquitously exists in the gene promoter region, Sox2 could act as a main transcription factor to regulate the expression of Cped1. Overexpressing Sox2 to detect the trans-regulatory effects of transcription factors, we found that Cped1 expression was significantly reduced (Figure 4I). Above results indicated that histone acetylation could co-operate with Sox2 in regulating Cped1 expression.

## Discussion

Recently, increasingly evidences showed that multiple factors may regulate the proliferation and differentiation of SSCs [18]. These factors were mainly divided into two big categories: (1) promote the proliferation of SSCs: growth promotion factor, GDNF, Lin28a, ID4 and plzf [19–23]; (2) promote the differentiation of SSCs: C-kit, Sohlh2, and mTORC1 [24,25]. Although genes regulated in the proliferation and differentiation have been studied, the key gene on the differentiation of ESCs into SSCs is still an emerging field. Our previous studies suggested that Cped1 significantly expressed in SSCs by RNA-seq of ESCs, PGCs, and SSCs. We speculated that Cped1 may have an important role in the regulation of SSCs. Bioinformatics analysis showed that Cped1 was specifically expressed in poultry and the function of Cped1 was unknown. Unlike markers such as integrin α6, β1, CD9, and CD24, Cped1 participates in the formation of SSCs and is only specifically expressed in poultry SSCs. Therefore, we explored the role of Cped1 in the formation of chicken SSCs to lay the foundation for the study of its function. We targeted Cped1 by CRISPR/Cas9 system. By knockouting Cped1, markers integrin α6 and integrin β1 were decreased *in vivo* and *in vitro*. In addition, the amount of SSCs was reduced, which was differentiated from ESCs. These results indicated that Cped1 is an important gene that promotes the formation of chicken SSCs.

By bioinformatics analysis, a Sox2-binding site was found in the active control area of Cped1 (−296 to −1 bp). After mutating the binding site of Sox2, the transcriptional activity of Cped1 increased while decreased when overexpressing Sox2. Above results suggested that Sox2 could negatively regulate the expression of Cped1. During the formation of SSCs, the deficiency of transcription factor Sox2 facilitates the expression of Cped1 and promotes the formation of SSCs. These results indirectly evidenced that Sox2 plays an important role in maintaining cell pluripotency and inhibiting cell differentiation as previous studies found. Since we did not find Oct4-binding sites in Cped1 promoter by bioinformatics analysis, we did not investigate whether Cped1 could be regulated by Oct4 cooperated with Sox2 [26]. This will be further studied.

**Figure 5:**
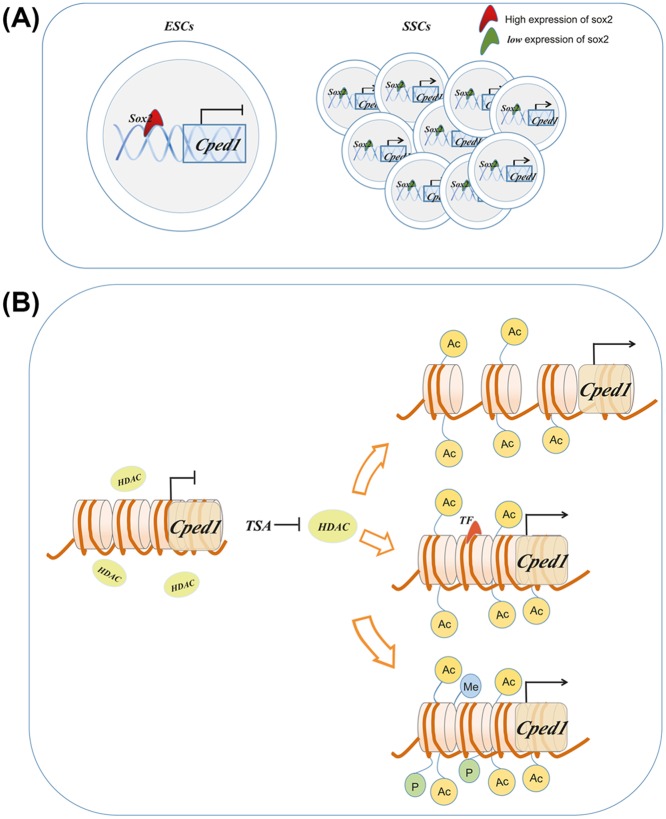
Mechanisms of histone acetylation and Sox2 in regulating the expression of Cped1 (**A**) Sox2 negatively regulates the expression of Cped1. (**B**) Histone acetylation may up-regulate the expression of Cped1 by three ways.

In addition to transcription factor, Cped1 may also be regulated by epigenetic modifications. DNA demethylation and histone acetylation were respectively added in the DF1 cells. The results implied that Cped1 is regulated by histone acetylation. Adding Histone deacetylase inhibitor TSA, the activity of promoter would increase. However, the activity of promoter would not change after adding 5-azaeytidi. The results in DF1 cells are as the same as in SSCs. Taken together, histone acetylation is involved in the regulation of Cped1 expression. According to the results, we established a Cped1 regulatory mechanism model: Cped1 continuously increased during the differentiation of ESCs to SSCs. The transcription factor Sox2 and histone acetylation participate in the regulation of Cped1 gene expression. Sox2 expresses mostly in ESCs and inhibits the expression of Cped1, which blocked the differentiation of ESCs. During the development, the expression of Sox2 gradually decreased, weakening the negative regulation of Cped1. Then its transcriptional activity was enhanced, which promotes the formation of SSCs (Figure 5A). Adding deacetylase inhibitors in SSCs increased the transcriptional activity of Cped1 and facilitates the formation of SSCs. There are three mechanisms of histone acetylation in regulating Cped1: (1) The acetylation of histones will weaken the interaction with DNA, loosen the chromatin, and facilitate the transcription. (2) Histone acetylation can provide specific sites for Cped1 recruiting transcription factors. (3) Histone acetylation can combine with other histone modifications (methylation and ubiquitination) to affect the transcription of Cped1 (Figure 5B). In the present study, we found histone acetylation could promote the transcription of Cped1. However, the specific regulatory models need to be further studied.

## Conclusion

In the present study, we found that Cped1 can promote the formation of chicken SSCs under the regulation of Sox2 and epigenetic modification of histone acetylation, which provides a solid foundation to study gene regulatory networks of chicken SSCs.

## Supporting information

## Abbreviations

Cped1: cadherin-like and PC-esterase domain containing 1
DMEM: Dulbecco’s modified eagle medium
ESC: embryonic stem cell
FBS: fetal bovine serum
PGC: primordial germ cell
qRT-PCR: quantitative reverse transcription-polymerase chain reaction
RA: retinoic acid
RNA-seq: RNA sequencing
SSC: spermatogonial stem cell
