# Supplementary figures and images for: Cped1 promotes chicken SSCs formation with the aid of histone acetylation and transcription factor Sox2
Source: Biosci Rep. 2018 Sep 14;38(5):BSR20180707. doi: 10.1042/BSR20180707 (PMC6137251; doi:10.1042/BSR20180707)

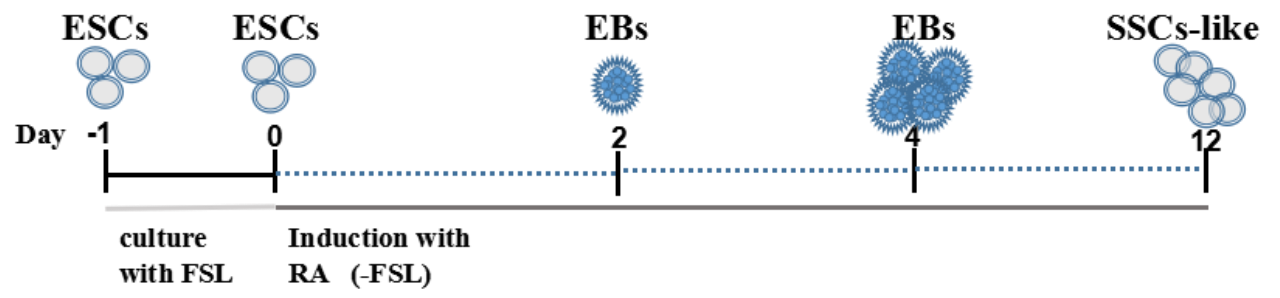

Supplement: Supplementary file 1 [file bsr20180707_Supp1.pdf]
